# Tetracarbon­ylbis(η^5^-cyclo­penta­dien­yl)bis(diphenyl­phosphine)dimolybdenum(*Mo*—*Mo*) hexane solvate

**DOI:** 10.1107/S1600536808017996

**Published:** 2008-06-19

**Authors:** Ginger V. Shultz, Stephanie A. Bossé, Lev N. Zakharov, David R. Tyler

**Affiliations:** a Department of Chemistry, 1253 University of Oregon, Eugene, Oregon 97403-1253, USA

## Abstract

The title compound, [Mo_2_(C_5_H_5_)_2_(C_12_H_11_P)_2_(CO)_4_]·C_6_H_14_, is a centrosymmetric Mo complex in which two Mo atoms are connected by an Mo—Mo bond [3.2072 (12) Å]. Each Mo atom is coordinated by an η^5^-cyclo­penta­dienyl ligand, two carbonyl ligands and a diphenyl­phosphine ligand in a piano-stool fashion.

## Related literature

For related literature, see: Adams *et al.* (1997 ▶); Chen *et al.* (2004 ▶); Daglen *et al.* (2007 ▶); Shultz *et al.* (2008 ▶); Tenhaeff & Tyler (1991 ▶); Tyler (2003 ▶); Van der Sluis & Spek (1990 ▶); Wilson & Shoemaker (1957 ▶).
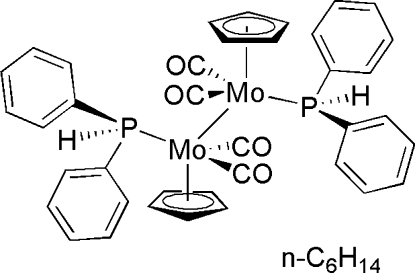


## Experimental

### 

#### Crystal data

[Mo_2_(C_5_H_5_)_2_(C_12_H_11_P)_2_(CO)_4_]·C_6_H_14_
*M* *_r_* = 892.63
Triclinic, 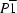

*a* = 8.6261 (18) Å
*b* = 9.2910 (19) Å
*c* = 13.697 (3) Å
α = 81.893 (4)°
β = 71.985 (4)°
γ = 73.896 (4)°
*V* = 1001.1 (4) Å^3^
*Z* = 1
Mo *K*α radiation
μ = 0.75 mm^−1^
*T* = 173 (2) K
0.15 × 0.07 × 0.01 mm

#### Data collection

Bruker SMART APEX CCD area-detector diffractometer
Absorption correction: multi-scan (*SADABS*; Sheldrick, 1995 ▶) *T*_min_ = 0.896, *T*_max_ = 0.993
11167 measured reflections
4330 independent reflections
2766 reflections with *I* > 2σ(*I*)
*R*_int_ = 0.086

#### Refinement

*R*[*F* ^2^ > 2σ(*F* ^2^)] = 0.066
*wR*(*F* ^2^) = 0.152
*S* = 0.95
4330 reflections
212 parameters
H atoms treated by a mixture of independent and constrained refinement
Δρ_max_ = 1.06 e Å^−3^
Δρ_min_ = −1.09 e Å^−3^

### 

Data collection: *SMART* (Bruker, 2000 ▶); cell refinement: *SAINT* (Bruker, 2000 ▶); data reduction: *SAINT*; program(s) used to solve structure: *SHELXTL* (Sheldrick, 2008 ▶); program(s) used to refine structure: *SHELXTL*; molecular graphics: *SHELXTL*; software used to prepare material for publication: *SHELXTL*.

## Supplementary Material

Crystal structure: contains datablocks I, global. DOI: 10.1107/S1600536808017996/hg2402sup1.cif

Structure factors: contains datablocks I. DOI: 10.1107/S1600536808017996/hg2402Isup2.hkl

Additional supplementary materials:  crystallographic information; 3D view; checkCIF report

## Figures and Tables

**Fig. 1.**
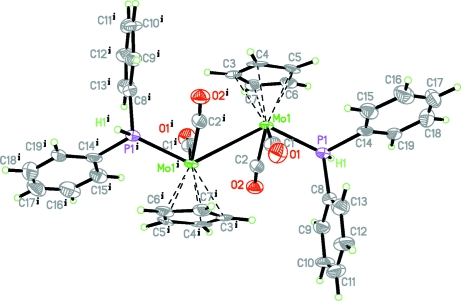
The structure of [Mo(CO)2(η5-C5H5)PHPh2]2 with 50% probability displacement ellipsoids and the atom-numbering scheme. [Symmetry code (i): -x,-y,-z].

## supplementary crystallographic information

### Comment

We previously reported the synthesis of photodegradable polymers that contain metal-metal bonds along the main chain (Tenhaeff & Tyler, 1991; Tyler, 2003). The metal-metal bond provides a convenient spectroscopic handle for monitoring the effect of external parameters as tensile stress (Chen *et al.*, 2004) and temperature (Daglen *et al.*, 2007) on the rate and onset of polymer backbone degradation. Recent work describes the preparation of phosphine-substituted dimeric molydenum complexes as precursors for step polymerization (Shultz *et al.*, 2008). The title complex [MoCp(CO)_2_(PPh_2_H)]_2_(hexane) was obtained in our attempts to prepare the [MoCp(CO)_2_(Ph_2_P(CH_2_)_3_C≡CH)]_2_ complex for polymerization. Attempts to grow single crystals of the last complex were unsuccessful and instead yielded crystals of the [MoCp(CO)_2_(PPh_2_H)]_2_(hexane). The synthesis of the [MoCp(CO)_2_(PPh_2_H)]_2_ was previously reported (Adams *et al.*, 1997), but the crystal structure has not been determined.

The compound [Mo(CO)_2_(η^5^-C_5_H_5_)PHPh_2_]_2_(C_6_H_14_) is a centrosymmetric Mo complex in which two Mo atoms are connected by a Mo—Mo bond. Each Mo atom is coordinated to an η^5^-cyclopentdienyl ligand, two carbonyl ligands, and a diphenylphosphine ligand in a piano-stool fashion (Fig. 1). The Mo—Mo bond length of 3.2072 (12) Å found in [Mo(CO)_2_(η^5^-C_5_H_5_)PHPh_2_]_2_ is within the range of single Mo—Mo bond lengths found in other related dimeric molybdenum complexes such as [MoCp(CO)_2_]_2_ (Wilson & Shoemaker, 1957) and [MoCp(CO)_2_(Ph_2_P(CH_2_)_6_CH═CH_2_)]_2_ (Shultz *et al.*, 2008). The solvent hexane molecule in the crystal structure is disordered around an inversion center.

### Experimental

The synthesis of [Mo(CO)_2_(η^5^-C_5_H_5_)PHPh_2_]_2_ was carried out by reaction of [CpMo(CO)_2_]_2_ with 2 equivalents of phosphine ligand Ph_2_P(CH_2_)_3_C≡CH, which contained a small amount of Ph_2_PH, in a diglyme solution at room temperature. Crystals suitable for X-ray analysis were grown by slow cooling in a diglyme/hexanes solution. Although [MoCp(CO)_2_Ph_2_P(CH_2_)_3_CHδb CH_2_]_2_ was the primary product of the reaction, only crystals of [Mo(CO)_2_(η^5^-C_5_H_5_)PHPh_2_]_2_(C_6_H_14_) were obtained.

### Refinement

The structure was solved using direct methods and refined with anisotropic thermal parameters for non-H atoms. Position of the H atom coordinated to the P atom was found from the residual density and this H atom was refined with isotropic thermal parameters. Other H atoms were positioned geometrically and refined in a rigid group model, C—H = 1.00 Å (Cp-ring) and 0.95 Å (Ph-rings); *U*_iso_(H) = 1.2*U*_eq_(C).

A highly disordered solvent molecule, most probably C_6_H_14_, was found to be present in crystal nearby an inversion center; however our attempts to locate the individual atoms were unsuccessful. Therefore, in order to take into account the contribution of the disordered solvent we applied, the solvent was treated by SQUEEZE technique (Van der Sluis & Spek, 1990). Correction of the X-ray data by SQUEEZE (56 electrons/cell) was close to the required value for one C_6_H_14_ molecule per the full unit cell (50 electrons/cell).

### Crystal data

**Table:**

[Mo_2_(C_5_H_5_)_2_(C_12_H_11_P)_2_(CO)_4_]·C_6_H_14_	*Z* = 1
*M**_r_* = 892.63	*F*_000_ = 456
Triclinic, *P*1	*D*_x_ = 1.481 Mg m^−^^3^
Hall symbol: -P 1	Mo *K*α radiation λ = 0.71073 Å
*a* = 8.6261 (18) Å	Cell parameters from 882 reflections
*b* = 9.2910 (19) Å	θ = 2.6–17.6º
*c* = 13.697 (3) Å	µ = 0.75 mm^−^^1^
α = 81.893 (4)º	*T* = 173 (2) K
β = 71.985 (4)º	Block, red
γ = 73.896 (4)º	0.15 × 0.07 × 0.01 mm
*V* = 1001.1 (4) Å^3^	

### Data collection

**Table:**

Bruker SMART APEX CCD area-detector diffractometer	4330 independent reflections
Radiation source: fine-focus sealed tube	2766 reflections with *I* > 2σ(*I*)
Monochromator: graphite	*R*_int_ = 0.086
*T* = 173(2) K	θ_max_ = 27.0º
φ and ω scans	θ_min_ = 1.6º
Absorption correction: multi-scan(SADABS; Sheldrick, 1995)	*h* = −10→10
*T*_min_ = 0.896, *T*_max_ = 0.993	*k* = −11→11
11167 measured reflections	*l* = −17→17

### Refinement

**Table:**

Refinement on *F*^2^	Secondary atom site location: difference Fourier map
Least-squares matrix: full	Hydrogen site location: inferred from neighbouring sites
*R*[*F*^2^ > 2σ(*F*^2^)] = 0.066	H atoms treated by a mixture of independent and constrained refinement
*wR*(*F*^2^) = 0.152	*w* = 1/[σ^2^(*F*_o_^2^) + (0.0637*P*)^2^] where *P* = (*F*_o_^2^ + 2*F*_c_^2^)/3
*S* = 0.95	(Δ/σ)_max_ < 0.001
4330 reflections	Δρ_max_ = 1.06 e Å^−^^3^
212 parameters	Δρ_min_ = −1.09 e Å^−^^3^
Primary atom site location: structure-invariant direct methods	Extinction correction: none

### Special details

**Table:**

Geometry. All e.s.d.'s (except the e.s.d. in the dihedral angle between two l.s. planes)are estimated using the full covariance matrix. The cell e.s.d.'s are takeninto account individually in the estimation of e.s.d.'s in distances, anglesand torsion angles; correlations between e.s.d.'s in cell parameters are onlyused when they are defined by crystal symmetry. An approximate (isotropic)treatment of cell e.s.d.'s is used for estimating e.s.d.'s involving l.s. planes.
Refinement. Refinement of *F*^2^ against ALL reflections. The weighted *R*-factor *wR* and goodness of fit *S* are based on *F*^2^, conventional *R*-factors *R* are based on *F*, with *F* set to zero for negative *F*^2^. The threshold expression of *F*^2^ > σ(*F*^2^) is used only for calculating *R*-factors(gt) *etc*. and is not relevant to the choice of reflections for refinement. *R*-factors based on *F*^2^ are statistically about twice as large as those based on *F*, and *R*- factors based on ALL data will be even larger.

### Fractional atomic coordinates and isotropic or equivalent isotropic displacement parameters (Å^2^)

**Table:**

	*x*	*y*	*z*	*U*_iso_*/*U*_eq_	
Mo1	0.08453 (7)	0.07667 (6)	0.06104 (5)	0.02499 (19)	
P1	0.2686 (2)	−0.04682 (18)	0.16299 (13)	0.0285 (4)	
O1	−0.1657 (6)	−0.0706 (6)	0.2330 (4)	0.0454 (13)	
O2	0.3623 (6)	−0.1648 (5)	−0.0744 (4)	0.0397 (12)	
C1	−0.0688 (9)	−0.0224 (8)	0.1649 (5)	0.0343 (16)	
C2	0.2458 (8)	−0.0818 (7)	−0.0233 (5)	0.0304 (15)	
C3	−0.0039 (10)	0.3062 (6)	−0.0307 (6)	0.0365 (18)	
H3A	−0.0649	0.3205	−0.0841	0.044*	
C4	−0.0793 (9)	0.3252 (7)	0.0762 (6)	0.0415 (19)	
H4A	−0.2028	0.3570	0.1110	0.050*	
C5	0.0493 (9)	0.3080 (7)	0.1222 (6)	0.0372 (18)	
H5A	0.0335	0.3272	0.1951	0.045*	
C6	0.2038 (10)	0.2748 (7)	0.0456 (6)	0.0375 (17)	
H6A	0.3164	0.2677	0.0548	0.045*	
C7	0.1708 (10)	0.2767 (7)	−0.0493 (6)	0.0401 (18)	
H7A	0.2562	0.2673	−0.1183	0.048*	
C8	0.2740 (9)	−0.2437 (7)	0.2043 (5)	0.0333 (16)	
C9	0.3917 (9)	−0.3568 (8)	0.1447 (6)	0.0393 (18)	
H9A	0.4724	−0.3321	0.0842	0.047*	
C10	0.3905 (11)	−0.5048 (8)	0.1739 (7)	0.051 (2)	
H10A	0.4710	−0.5822	0.1334	0.061*	
C11	0.2754 (11)	−0.5405 (9)	0.2598 (7)	0.055 (2)	
H11A	0.2769	−0.6431	0.2791	0.066*	
C12	0.1571 (11)	−0.4323 (8)	0.3193 (6)	0.051 (2)	
H12A	0.0756	−0.4580	0.3790	0.061*	
C13	0.1593 (10)	−0.2850 (8)	0.2902 (5)	0.0435 (19)	
H13A	0.0780	−0.2088	0.3313	0.052*	
C14	0.2585 (9)	0.0315 (7)	0.2804 (5)	0.0323 (16)	
C15	0.1047 (10)	0.0952 (8)	0.3460 (5)	0.0408 (18)	
H15A	0.0041	0.1036	0.3284	0.049*	
C16	0.0974 (12)	0.1476 (9)	0.4391 (6)	0.056 (2)	
H16A	−0.0087	0.1892	0.4853	0.067*	
C17	0.2421 (14)	0.1391 (9)	0.4635 (7)	0.063 (3)	
H17A	0.2370	0.1752	0.5263	0.076*	
C18	0.3954 (13)	0.0778 (9)	0.3962 (7)	0.059 (2)	
H18A	0.4960	0.0736	0.4125	0.071*	
C19	0.4053 (10)	0.0227 (7)	0.3057 (6)	0.0412 (19)	
H19A	0.5119	−0.0211	0.2608	0.049*	
H1	0.427 (7)	−0.057 (6)	0.119 (4)	0.017 (14)*	

### Atomic displacement parameters (Å^2^)

**Table:**

	*U*^11^	*U*^22^	*U*^33^	*U*^12^	*U*^13^	*U*^23^
Mo1	0.0325 (3)	0.0102 (3)	0.0349 (3)	−0.0046 (2)	−0.0133 (2)	−0.0028 (2)
P1	0.0351 (10)	0.0174 (9)	0.0347 (10)	−0.0047 (7)	−0.0128 (8)	−0.0041 (7)
O1	0.045 (3)	0.049 (3)	0.042 (3)	−0.015 (3)	−0.011 (3)	0.004 (3)
O2	0.037 (3)	0.030 (3)	0.047 (3)	0.000 (2)	−0.010 (2)	−0.011 (2)
C1	0.031 (4)	0.030 (4)	0.038 (4)	0.000 (3)	−0.010 (3)	−0.005 (3)
C2	0.032 (4)	0.028 (4)	0.038 (4)	−0.016 (3)	−0.013 (3)	0.002 (3)
C3	0.060 (5)	0.004 (3)	0.051 (5)	−0.010 (3)	−0.027 (4)	0.007 (3)
C4	0.036 (4)	0.005 (3)	0.078 (6)	0.002 (3)	−0.014 (4)	−0.008 (3)
C5	0.049 (5)	0.011 (3)	0.060 (5)	−0.005 (3)	−0.025 (4)	−0.012 (3)
C6	0.048 (5)	0.015 (3)	0.055 (5)	−0.016 (3)	−0.018 (4)	−0.001 (3)
C7	0.061 (5)	0.017 (4)	0.045 (5)	−0.014 (3)	−0.018 (4)	0.005 (3)
C8	0.042 (4)	0.022 (4)	0.039 (4)	−0.004 (3)	−0.021 (3)	−0.001 (3)
C9	0.034 (4)	0.029 (4)	0.060 (5)	−0.006 (3)	−0.021 (4)	−0.005 (3)
C10	0.058 (5)	0.015 (4)	0.083 (6)	0.006 (4)	−0.037 (5)	−0.010 (4)
C11	0.073 (6)	0.026 (4)	0.083 (7)	−0.017 (4)	−0.048 (6)	0.012 (4)
C12	0.074 (6)	0.026 (4)	0.054 (5)	−0.016 (4)	−0.021 (5)	0.007 (4)
C13	0.059 (5)	0.036 (4)	0.034 (4)	−0.012 (4)	−0.011 (4)	0.000 (3)
C14	0.049 (5)	0.011 (3)	0.038 (4)	−0.010 (3)	−0.013 (3)	0.002 (3)
C15	0.049 (5)	0.033 (4)	0.040 (4)	−0.016 (4)	−0.007 (4)	−0.002 (3)
C16	0.088 (7)	0.041 (5)	0.038 (5)	−0.025 (5)	−0.009 (5)	−0.003 (4)
C17	0.119 (9)	0.041 (5)	0.047 (5)	−0.026 (5)	−0.043 (6)	−0.001 (4)
C18	0.084 (7)	0.048 (5)	0.061 (6)	−0.015 (5)	−0.046 (6)	0.002 (4)
C19	0.061 (5)	0.019 (4)	0.057 (5)	−0.012 (3)	−0.035 (4)	0.002 (3)

### Geometric parameters (Å, °)

**Table:**

Mo1—C1	1.940 (8)	C7—H7A	1.0000
Mo1—C2	1.946 (7)	C8—C13	1.369 (9)
Mo1—C6	2.302 (6)	C8—C9	1.393 (9)
Mo1—C5	2.324 (6)	C9—C10	1.379 (10)
Mo1—C4	2.349 (6)	C9—H9A	0.9500
Mo1—C7	2.360 (7)	C10—C11	1.353 (11)
Mo1—C3	2.376 (6)	C10—H10A	0.9500
Mo1—P1	2.3866 (18)	C11—C12	1.365 (11)
Mo1—Mo1^i^	3.2072 (12)	C11—H11A	0.9500
P1—C14	1.826 (7)	C12—C13	1.374 (10)
P1—C8	1.829 (7)	C12—H12A	0.9500
P1—H1	1.29 (5)	C13—H13A	0.9500
O1—C1	1.173 (8)	C14—C15	1.376 (9)
O2—C2	1.176 (7)	C14—C19	1.391 (9)
C3—C7	1.401 (10)	C15—C16	1.406 (10)
C3—C4	1.421 (10)	C15—H15A	0.9500
C3—H3A	1.0000	C16—C17	1.369 (12)
C4—C5	1.399 (9)	C16—H16A	0.9500
C4—H4A	1.0000	C17—C18	1.378 (12)
C5—C6	1.404 (10)	C17—H17A	0.9500
C5—H5A	1.0000	C18—C19	1.376 (10)
C6—C7	1.411 (9)	C18—H18A	0.9500
C6—H6A	1.0000	C19—H19A	0.9500
			
C1—Mo1—C2	105.4 (3)	Mo1—C4—H4A	125.8
C1—Mo1—C6	138.8 (3)	C4—C5—C6	108.1 (7)
C2—Mo1—C6	109.0 (3)	C4—C5—Mo1	73.5 (4)
C1—Mo1—C5	106.1 (3)	C6—C5—Mo1	71.5 (4)
C2—Mo1—C5	143.9 (3)	C4—C5—H5A	125.8
C6—Mo1—C5	35.3 (2)	C6—C5—H5A	125.8
C1—Mo1—C4	99.1 (3)	Mo1—C5—H5A	125.8
C2—Mo1—C4	150.1 (3)	C5—C6—C7	108.2 (7)
C6—Mo1—C4	58.4 (3)	C5—C6—Mo1	73.2 (4)
C5—Mo1—C4	34.8 (2)	C7—C6—Mo1	74.7 (4)
C1—Mo1—C7	156.6 (3)	C5—C6—H6A	125.5
C2—Mo1—C7	95.6 (3)	C7—C6—H6A	125.5
C6—Mo1—C7	35.2 (2)	Mo1—C6—H6A	125.5
C5—Mo1—C7	58.3 (3)	C3—C7—C6	107.9 (7)
C4—Mo1—C7	57.9 (3)	C3—C7—Mo1	73.4 (4)
C1—Mo1—C3	123.8 (3)	C6—C7—Mo1	70.1 (4)
C2—Mo1—C3	115.2 (3)	C3—C7—H7A	126.0
C6—Mo1—C3	58.1 (2)	C6—C7—H7A	126.0
C5—Mo1—C3	58.1 (2)	Mo1—C7—H7A	126.0
C4—Mo1—C3	35.0 (2)	C13—C8—C9	117.9 (7)
C7—Mo1—C3	34.4 (2)	C13—C8—P1	122.1 (5)
C1—Mo1—P1	81.6 (2)	C9—C8—P1	120.0 (6)
C2—Mo1—P1	75.99 (19)	C10—C9—C8	119.7 (7)
C6—Mo1—P1	85.34 (19)	C10—C9—H9A	120.1
C5—Mo1—P1	91.73 (18)	C8—C9—H9A	120.1
C4—Mo1—P1	125.1 (2)	C11—C10—C9	120.3 (8)
C7—Mo1—P1	113.98 (19)	C11—C10—H10A	119.8
C3—Mo1—P1	143.43 (17)	C9—C10—H10A	119.8
C1—Mo1—Mo1^i^	73.9 (2)	C10—C11—C12	121.4 (7)
C2—Mo1—Mo1^i^	67.11 (18)	C10—C11—H11A	119.3
C6—Mo1—Mo1^i^	141.14 (18)	C12—C11—H11A	119.3
C5—Mo1—Mo1^i^	139.46 (17)	C11—C12—C13	118.0 (8)
C4—Mo1—Mo1^i^	104.62 (19)	C11—C12—H12A	121.0
C7—Mo1—Mo1^i^	106.02 (18)	C13—C12—H12A	121.0
C3—Mo1—Mo1^i^	87.55 (17)	C8—C13—C12	122.6 (7)
P1—Mo1—Mo1^i^	127.32 (5)	C8—C13—H13A	118.7
C14—P1—C8	102.5 (3)	C12—C13—H13A	118.7
C14—P1—Mo1	121.2 (2)	C15—C14—C19	119.8 (7)
C8—P1—Mo1	117.1 (2)	C15—C14—P1	119.8 (5)
C14—P1—H1	96 (2)	C19—C14—P1	120.3 (6)
C8—P1—H1	100 (2)	C14—C15—C16	119.5 (7)
Mo1—P1—H1	116 (2)	C14—C15—H15A	120.2
O1—C1—Mo1	173.7 (6)	C16—C15—H15A	120.2
O2—C2—Mo1	168.9 (5)	C17—C16—C15	120.5 (8)
C7—C3—C4	107.8 (7)	C17—C16—H16A	119.8
C7—C3—Mo1	72.2 (4)	C15—C16—H16A	119.8
C4—C3—Mo1	71.4 (4)	C16—C17—C18	119.3 (8)
C7—C3—H3A	126.0	C16—C17—H17A	120.3
C4—C3—H3A	126.0	C18—C17—H17A	120.3
Mo1—C3—H3A	126.0	C19—C18—C17	121.2 (8)
C5—C4—C3	108.0 (7)	C19—C18—H18A	119.4
C5—C4—Mo1	71.6 (4)	C17—C18—H18A	119.4
C3—C4—Mo1	73.5 (4)	C18—C19—C14	119.7 (8)
C5—C4—H4A	125.8	C18—C19—H19A	120.2
C3—C4—H4A	125.8	C14—C19—H19A	120.2

Symmetry codes: (i) −*x*, −*y*, −*z*.
